# Silencing hTERT attenuates cancer stem cell-like characteristics and radioresistance in the radioresistant nasopharyngeal carcinoma cell line CNE-2R

**DOI:** 10.18632/aging.104167

**Published:** 2020-11-24

**Authors:** Kaihua Chen, Ling Li, Song Qu, Xinbin Pan, Yongchu Sun, Fangzhu Wan, Binbin Yu, Lei Zhou, Xiaodong Zhu

**Affiliations:** 1 Department of Radiation Oncology, Guangxi Medical University Cancer Hospital, Nanning 530021, Guangxi, China; 2 Key Laboratory of Early Prevention and Treatment for Regional High Frequency Tumor (Guangxi Medical University), Ministry of Education, Nanning 530021, Guangxi, China; 3 Guangxi Key Laboratory of Early Prevention and Treatment for Regional High Frequency Tumor, Guangxi Medical University, Nanning 530021, Guangxi, China

**Keywords:** nasopharyngeal carcinoma, radioresistance, hTERT, cancer stem cells, RNA interference

## Abstract

Objective: This study aimed to explore the effect of silencing hTERT on the CSC-like characteristics and radioresistance of CNE-2R cells.

Results: Silencing hTERT suppressed CNE-2R cell proliferation and increased the cell apoptosis rate and radiosensitivity *in vitro*. Moreover, it could also inhibit the growth of xenografts and increase the apoptosis index and radiosensitivity *in vivo*. Further study discovered that after silencing hTERT, telomerase activity in CNE-2R cells was markedly suppressed, along with remarkably down-regulated stem cell-related protein levels both *in vitro* and *in vivo*.

Conclusion: Silencing hTERT can suppress the CSC-like characteristics of CNE-2R cells to enhance their radiosensitivity, revealing that hTERT may become a potential target for treating radioresistant NPC.

Methods: An RNAi lentiviral vector specific to the hTERT gene was constructed to infect CNE-2R cells, the hTERT silencing effect was verified through qPCR and Western blot assays, and telomerase activity was detected by PCR-ELISA. Moreover, radiosensitivity *in vitro* was detected through colony formation assays, CCK-8 assays and flow cytometry. Tumor growth and radioresistance were also evaluated using xenograft models, while the apoptosis index in xenografts was measured through TUNEL assay. Levels of stem cell-related proteins were determined *in vitro* and *in vivo*.

## INTRODUCTION

Nasopharyngeal carcinoma (NPC) is one of the most common malignancies of the head and neck in China, whose morbidity is second only to thyroid cancer [1]. Its epidemiological characteristic is its unique geographical distribution, which frequently occurs in areas such as South China [2]. Radiotherapy is a major treatment method and an essential part of radical treatment for NPC [3]. With continuous discoveries in radiotherapy and therapeutic schemes, the five-year overall survival rate of NPC has reached about 80% [4]. However, more than 20% of NPC patients develop recurrence or distant metastasis after standard treatment [5, 6]. Currently, radioresistance is considered the major obstacle for the effective treatment of NPC [7, 8], but the specific mechanism of radioresistance remains unclear. Therefore, exploring the potential mechanism of radioresistance in NPC will contribute to improving the radiosensitivity of NPC, thus improving the clinical efficacy.

In our previous study, the radioresistant cell line CNE-2R was successfully established based on the poorly differentiated NPC cell line CNE-2 using fractionated irradiation [9]. Further study discovered that CNE-2R cells displayed cancer stem cell (CSC)-like characteristics and marked telomerase activity, along with high expression of human telomerase reverse transcriptase (hTERT) [10]. Telomerase is highly expressed in CSCs [11, 12] and is required for self-renewal, progression and immortalization of CSCs [13]. hTERT is the essential catalytic subunit that maintains telomerase activity and regulates telomerase activity at the transcriptional level [14]. Down-regulation of hTERT transcription may be a potential mechanism for suppressing telomerase activity [15–17]. Some studies have indicated that downregulation of hTERT expression could eliminate the CSC phenotype [18–20]. Therefore, it was reasonable to consider that hTERT could regulate the CSC-like characteristics of CNE-2R cells.

Moreover, some scholars have discovered that interference with hTERT could affect the radiosensitivity of cervical cancer [21] and breast cancer [22]. On the other hand, inhibition of telomerase activity could also increase the radiosensitivity of various cancers [23–26]. Consequently, we speculated that high hTERT expression and telomerase activity was related to the radioresistance of CNE-2R cells, and down-regulation of hTERT expression might potentially inhibit telomerase activity and CSC-like characteristics, thus enhancing the radiosensitivity of CNE-2R cells.

In this study, we used lentiviral vector-mediated RNA interference (RNAi) technology to silence hTERT expression in CNE-2R cells and then detected changes in the radiosensitivity and CSC-like characteristics of CNE-2R cells before and after hTERT silencing. To the best of our knowledge, this study is the first to investigate the relationship between hTERT and NPC radioresistance, as well as the underlying mechanism, which might provide a potential molecular target for treating radioresistant NPC.

## RESULTS

### Effective silencing of hTERT in CNE-2R cells

**Figure 1:**
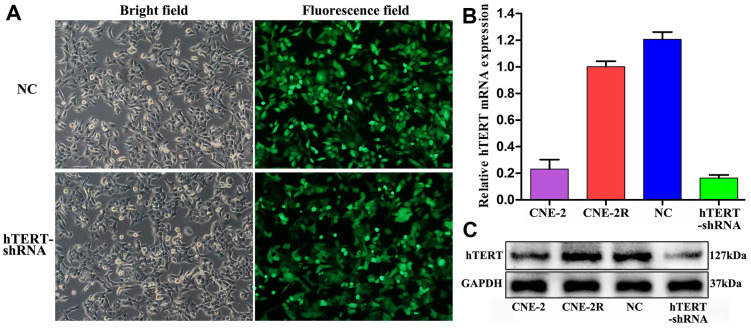
**Effective silencing of hTERT.** (**A**) CNE-2R cell infection rate observed under an inverted fluorescence microscope (200×); (**B**) hTERT mRNA expression detected by qPCR; (**C**) hTERT protein expression detected by Western blot assay.

After lentiviral infection of CNE-2R cells for 96 h, approximately 95% of cells showed green fluorescence under an inverted fluorescence microscope, and they were at favorable growth status (Figure 1A). The qPCR results indicated that the relative hTERT mRNA expression in hTERT-shRNA cells (0.164±0.023) was remarkably lower than that in NC (1.207±0.054) and CNE-2R cells (1.000±0.041) (P<0.001). Moreover, the results also verified that the relative expression in parent CNE-2 cells (0.231±0.071) was notably lower than that in CNE-2R cells (P<0.01) (Figure 1B). Similarly, Western blot results also suggested that hTERT protein expression in hTERT-shRNA cells was markedly down-regulated, which was consistent with the qPCR results (Figure 1C). The above findings indicated effective silencing of hTERT in CNE-2R cells; therefore, the cells could be used in subsequent functional assays.

### hTERT silencing enhanced radiosensitivity *in vitro*

**Figure 2:**
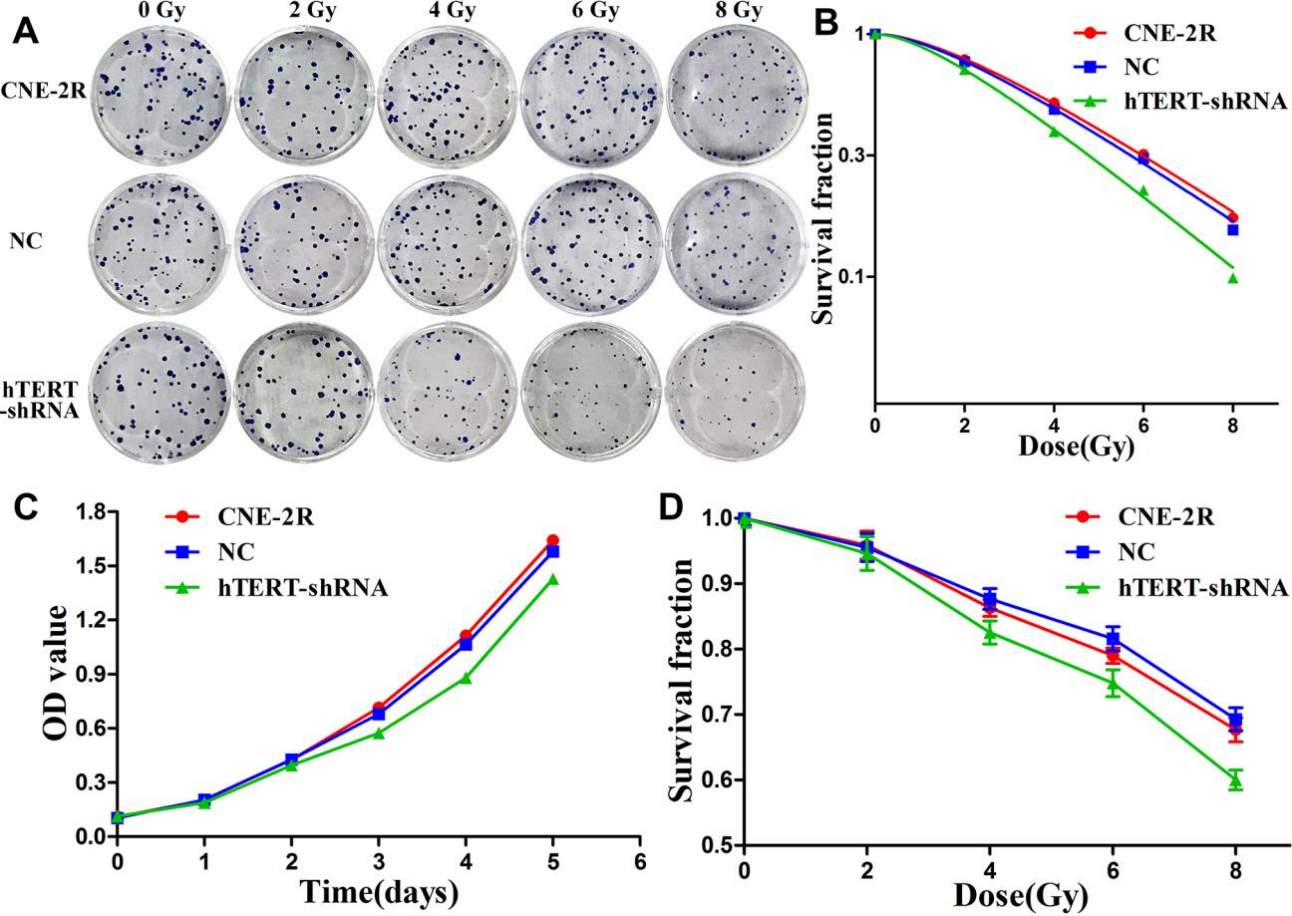
**hTERT silencing enhanced radiosensitivity.** (**A**) The radiosensitivity of CNE-2R, NC and hTERT-shRNA cells was compared through colony formation assay; (**B**) The dose-survival curves were fitted using the single-hit multi-target model; (**C**) Cell proliferation detected using CCK-8 assay; (**D**) The radiosensitivity was compared through the CCK-8 assay.

The radiosensitivity of CNE-2R cells, NC cells and hTERT-shRNA cells, was detected through a colony formation assay (Figure 2A). Then, the dose-survival curves were fitted using a single-hit multi-target model, and the results indicated that the survival fractions (SFs) of hTERT-shRNA cells at all radiation doses were lower than those of CNE-2R cells and NC cells (Figure 2B). The radiobiological parameters are displayed in Table 1. The sensitization enhancement ratio (SER) of hTERT-shRNA cells to CNE-2R cells was calculated as SER=D_0_ (CNE-2R)/D_0_ (hTERT-shRNA)=1.23>1, revealing that hTERT-shRNA cells were more sensitive to irradiation. Furthermore, the proliferation capacity and radiosensitivity of cells were detected through CCK-8 assay, and the results suggested that the proliferation of hTERT-shRNA cells was suppressed (P<0.05, Figure 2C). After irradiation at various doses, the SFs of hTERT-shRNA cells were markedly lower than those of CNE-2R cells and NC cells (P<0.01, Figure 2D).

**Table 1:** The radiobiological parameters in the single-hit multi-target model (Mean±SD).

**Cell lines**	**D_0_**	**D_q_**	**SF_2_**
CNE-2R	3.328±0.235	2.379±0.194	0.794±0.012
NC	3.343±0.105	2.145±0.240	0.782±0.030
hTERT-shRNA	2.831±0.119	1.723±0.149	0.724±0.020
*P*	0.009	0.018	0.017

**D_0_** was the mean lethal dose, which was theoretically the radiation dose required to hit each cell; **D_q_** was the quasi-threshold dose, which reflected the repair capacity of sublethal cell injury; and SF_2_ was the survival fraction at the dose of 2 Gy.

### hTERT silencing promoted cell apoptosis

**Figure 3:**
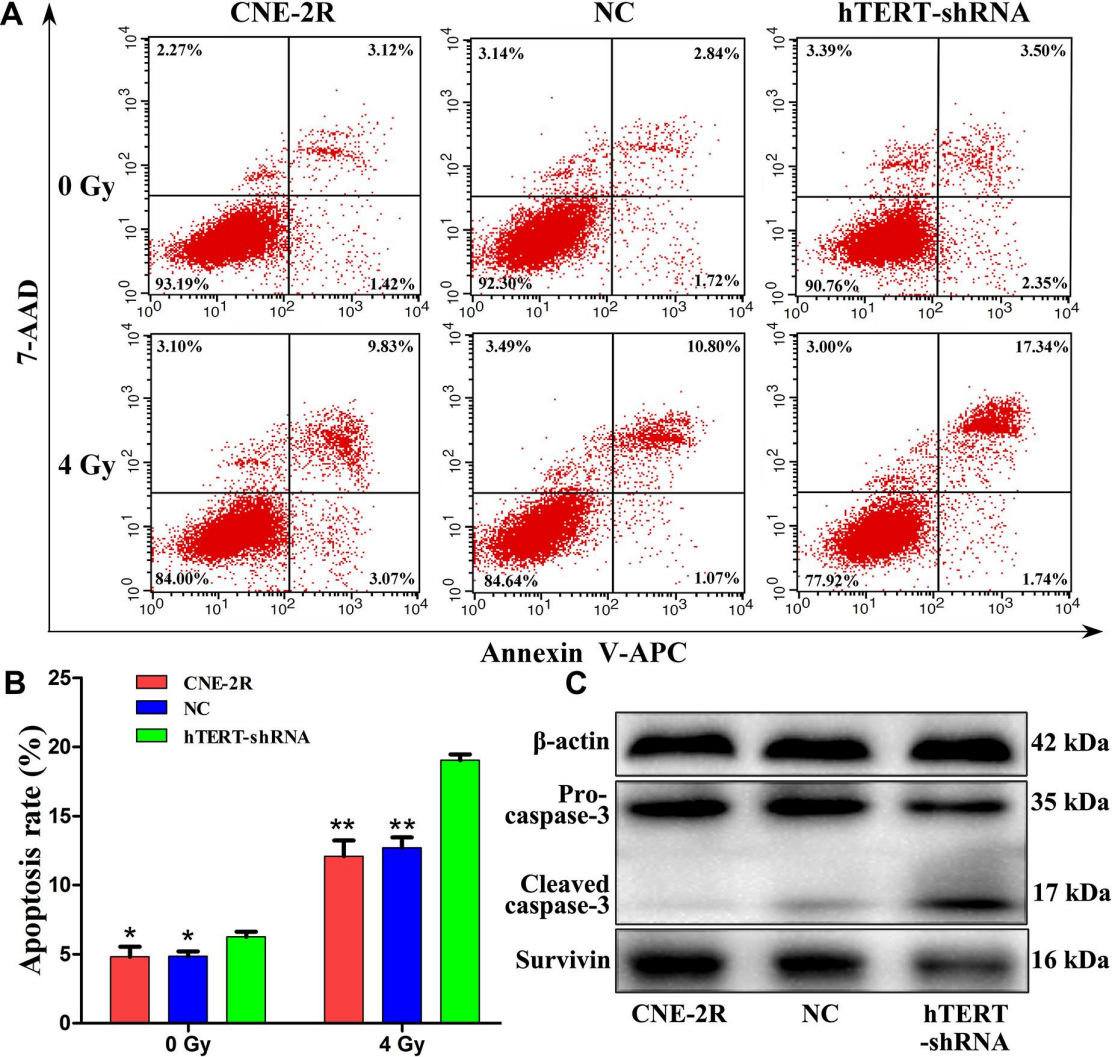
**hTERT silencing promoted apoptosis.** (**A**) The apoptosis rates after irradiation at 0 Gy and 4 Gy; (**B**) Histogram of the apoptosis rate in each group (* indicates P<0.05 compared with hTERT-shRNA cells, while ** indicates P<0.01 compared with hTERT-shRNA cells); (**C**) Expression of apoptosis-related proteins in each group after irradiation at 4 Gy.

Apoptosis and radiosensitivity changes in CNE-2R cells after hTERT silencing were detected through flow cytometry (Figure 3A). The results demonstrated that at the time of non-irradiation (0 Gy), the apoptosis rates of CNE-2R, NC and hTERT-shRNA cells were 4.82±0.73%, 4.85±0.35% and 6.25±0.38%, respectively (P=0.023). After irradiation at a dose of 4 Gy, the apoptosis rates of CNE-2R, NC and hTERT-shRNA cells were 12.10±1.14%, 12.71±0.74% and 19.03±0.43%, respectively (P<0.01). Differences in the apoptosis rates in CNE-2R and NC cells before and after irradiation were not statistically significant (P>0.05) (Figure 3B). Taken together, the apoptosis rates in hTERT-shRNA cells were more notablely increased than those in CNE-2R cells and NC cells after irradiation, suggesting that hTERT-shRNA cells were more sensitive to irradiation.

To further understand the role of hTERT in apoptosis after irradiation, the apoptosis-related proteins Survivin and Caspase-3 were detected. Briefly, the cells were exposed to 4 Gy X-ray. Forty-eight hours later, total proteins were extracted, and the expression of Survivin and Caspase-3 proteins was detected through Western blot assay. The results revealed that, compared with that in CNE-2R and NC cells, Survivin protein expression in hTERT-shRNA cells was remarkably down-regulated, while the Cleaved-caspase-3 protein expression was notably increased (Figure 3C).

### Changes in telomerase activity, β-catenin and stem cell-related proteins

**Figure 4:**
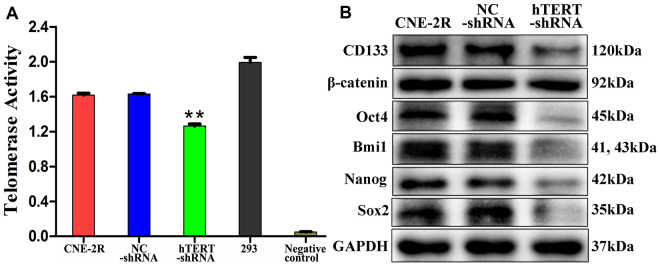
**hTERT silencing reduced telomerase activity and stem cell-related proteins expression *in vitro*.** (**A**) Telomerase activity detected utilizing the TRAP method (** indicates P<0.01 compared with CNE-2R cells); (**B**) Expression of β-catenin and stem cell-related proteins detected by Western blot assay.

Telomerase activities were detected utilizing the telomeric repeat amplification protocol (TRAP). The results suggested that, compared with that in NC cells (1.629±0.007) and CNE-2R cells (1.618±0.022), the telomerase activity in hTERT-shRNA cells (1.263±0.024) was obviously reduced (P<0.01, Figure 4A). To observe whether hTERT silencing can affect the CSC-like characteristics, the expression of β-catenin and stem cell-related proteins was detected through Western blot assay. The results revealed that the expression of Sox2, Bmi1, Nanog, Oct4 and CD133 in hTERT-shRNA cells was significantly lower than that in NC and CNE-2R cells, but the expression of β-catenin showed no significant difference among the three groups (Figure 4B).

### hTERT silencing enhanced radiosensitivity *in vivo*

**Figure 5:**
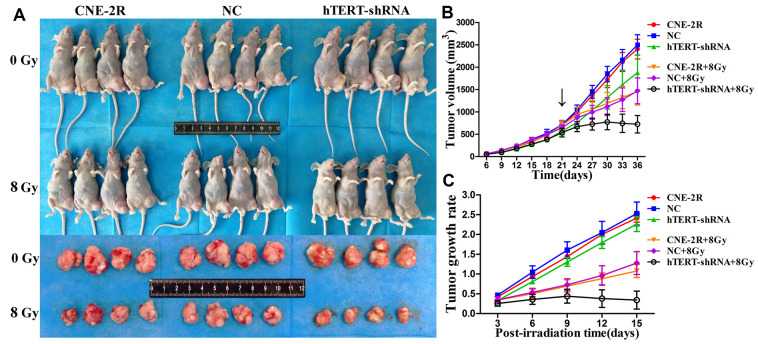
**Effect of hTERT silencing on tumor growth.** (**A**) Tumor sizes when cells were injected into nude mice and allowed to grow to day 36; (**B**) Growth curve of tumors in each group after receiving irradiation at 0 Gy or 8 Gy (black arrow indicates the time point of irradiation); (**C**) Tumor growth rate in each group after irradiation.

To further evaluate the effect of hTERT silencing on tumor growth, a xenograft model was constructed. Subcutaneous nodules could be palpable 3 days after cells injection. The tumor size was measured from day 6 and was recorded every 3 days. On day 21, the nude mice in each group were randomly divided into 2 subgroups, with 4 in each subgroup, to receive irradiation at 0 or 8 Gy. On day 36, all nude mice were sacrificed, and the tumors were collected (Figure 5A). The tumor growth curve indicated that when no irradiation was applied (0 Gy), the tumor volume in the hTERT-shRNA group at each time point was slightly smaller than those in the CNR-2R and NC groups (P<0.05). After irradiation at 8 Gy, the tumor growth in the three groups was inhibited, but the inhibition of the hTERT-shRNA group was more obvious (P<0.05) (Figure 5B). In addition, the tumor growth rates were further calculated, and the results showed that the tumor growth rate after irradiation in the hTERT-shRNA group at each time point was notably lower than that in the NC and CNE-2R groups (Figure 5C).

### hTERT silencing could induce apoptosis *in vivo*

**Figure 6:**
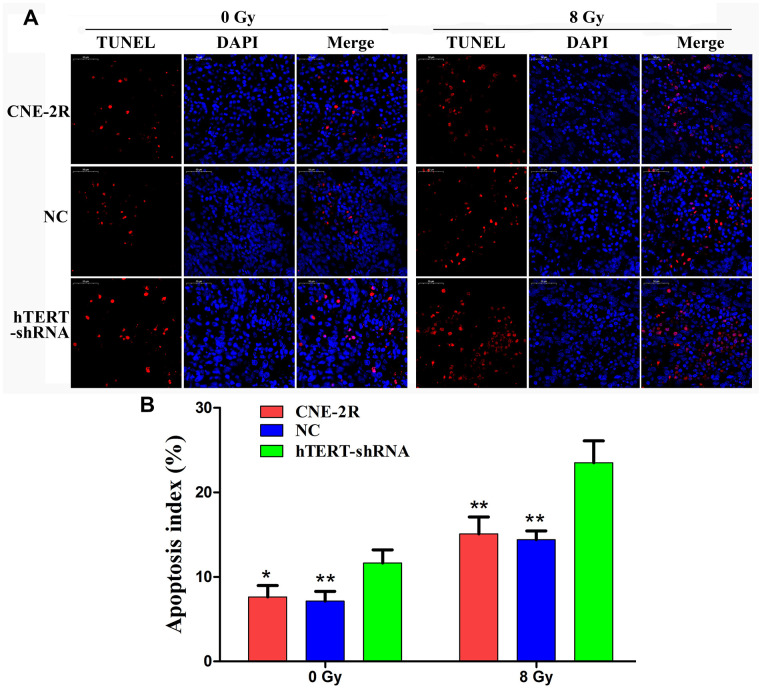
**hTERT silencing could induce apoptosis *in vivo*.** (**A**) Apoptotic cells in tumors detected by TUNEL, red fluorescence (TMR red) represents positive apoptotic cell nucleus, while blue fluorescence (DAPI) was used for nuclear localization (200×); (**B**) Histogram of the apoptosis index in each group (* indicates P<0.05 compared with the hTERT-shRNA group, and ** indicates P<0.01 compared with the hTERT-shRNA group).

Apoptotic cells in the tumors were detected through TUNEL assay (Figure 6A). The results indicated that when no irradiation was applied, the apoptosis indexes in the CNE-2R, NC and hTERT-shRNA groups were 7.63±1.33%, 7.13±1.15% and 11.64±1.54%, respectively (P<0.05). After radiation at 8 Gy, the apoptosis indexes in the CNE-2R, NC and hTERT-shRNA groups were 15.08±2.00%, 14.38±1.06% and 23.50±2.58%, respectively (P<0.01). Moreover, the difference in the apoptotic index between the CNE-2R and NC groups was not statistically significant (P>0.05) (Figure 6B). Taken together, the above findings indicated that after radiation at 8 Gy, the apoptosis index in the hTERT-shRNA group was more markedly increased than that in the CNE-2R and NC groups, revealing that tumors in the hTERT-shRNA group were more sensitive to irradiation.

### Expression of hTERT, β-catenin and stem cell-related proteins *in vivo*

**Figure 7:**
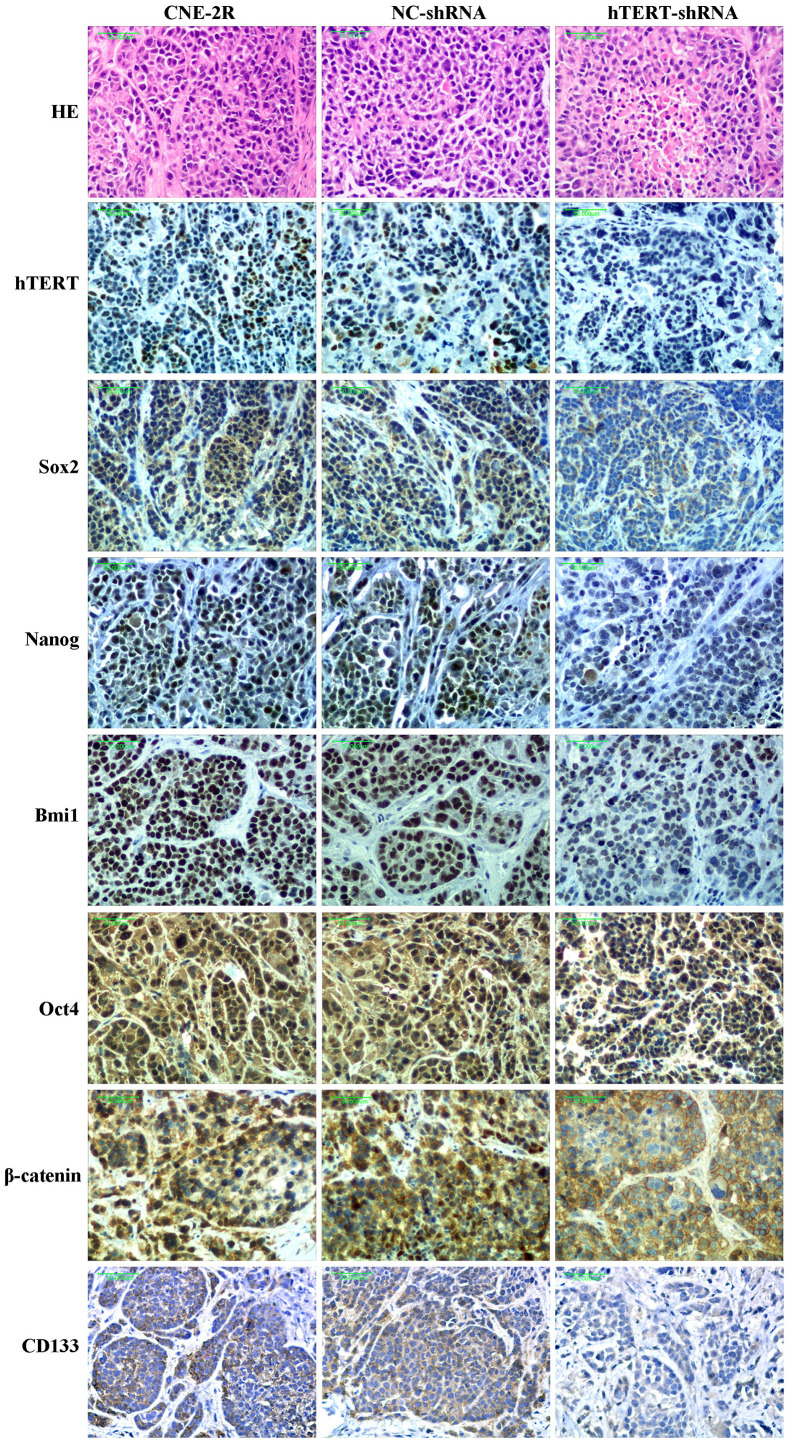
**Histological morphology and expression of hTERT, Sox2, Nanog, Bmi1, Oct4, CD133 and β-catenin proteins in each group (200×).**

The histological morphology of the tumor was observed through HE staining, and the results showed that the necrotic area in the hTERT-shRNA group was slightly increased. hTERT protein expression in tumors was detected by IHC, and the results revealed that hTERT protein expression in the hTERT-shRNA group was remarkably lower than that in the CNE-2R and NC groups. Thus, it could be figured out that, lentiviral vector-mediated hTERT interference could be stably inherited and effectively expressed *in vivo*. In order to preliminarily understand whether hTERT silencing would also affect the CSC-like characteristics of CNE-2R cells *in vivo*, the expression of β-catenin and stem cell-related proteins in tumors was detected. The results indicated that the expression of Sox2, Bmi1, Nanog, Oct4 and CD133 proteins in the hTERT-shRNA group was significantly lower than that in the CNE-2R and NC groups. The expression of β-catenin protein was strongly positive in the three groups, but it was mainly expressed in the membrane and cytoplasm in the hTERT-shRNA group, and expression could also be observed in part of nuclei in the CNE-2R and NC groups (Figure 7).

## DISCUSSION

In recent years, the role of hTERT in human disease, especially in tumors, has attracted wide attention. In normal human cells, hTERT is generally less or not expressed, except for embryonic stem cells (ESCs) and germ cells; however, it is highly expressed in all malignant human tumors. It is thought to maintain the immortalization of cancer cells [27] and is closely related to tumor transformation, growth and metastasis [28]. Previous studies have suggested that interfering with hTERT expression is an effective method for targeted therapy of tumors [27–30]. Moreover, some studies have reported that hTERT is related to the radiosensitivity of cervical cancer and breast cancer, but the mechanism remains unclear [21, 22]. In our previous study, the radioresistant NPC cell line CNE-2R had been established [9]. Further study discovered that hTERT gene and protein expression in CNE-2R cells was markedly higher than that in parent CNE-2 cells, revealing that high hTERT expression might be related to NPC radioresistance [10]. This study aimed to explore the relationship between hTERT and NPC radioresistance as well as the potential mechanism in CNE-2R cells.

Currently, some studies have indicated that, down-regulating hTERT could suppress the proliferation and promote apoptosis of various tumor cells, including NPC cells [29, 31–35]. In this study, we adopted lentiviral vector-mediated RNAi technology to silence hTERT in CNE-2R cells and obtained stable hTERT-shRNA cells with favorable silencing effects. The results of the CCK-8 assay and flow cytometry also demonstrated that hTERT silencing could suppress proliferation and promote apoptosis. After irradiation at 4 Gy, the results of flow cytometry revealed that the apoptosis rates in the three groups were markedly increased, but those in the hTERT-shRNA group were more strongly increased. In addition, it was also discovered that after irradiation at 4 Gy, the expression of the apoptosis-related protein Survivin in hTERT-shRNA cells was significantly down-regulated, while that of Cleaved-caspase 3 protein was up-regulated. Moreover, it was further confirmed through colony formation assay and CCK-8 assay that hTERT-shRNA cells were more sensitive to irradiation. The above results demonstrated that hTERT silencing could enhance the radiosensitivity of CNE-2R cells *in vitro*. Our research results were similar to those by Zhang et al., which indicated that down-regulating hTERT could enhance the radiosensitivity of SiHa cells [21].

Specific genes silenced through lentivirus-mediated RNAi technology can be stably inherited and effectively expressed in multiple xenograft models [36–40]. Our previous study had also successfully constructed a stable cofilin-2 silencing NPC xenograft model [41]. This study also supported the above finding, and the IHC results revealed that tumors formed by hTERT-shRNA cells expressed low levels of the hTERT protein. Zhao et al. found that silencing hTERT could promote apoptosis in oral squamous carcinoma xenografts and suppress tumor growth [32]. In this study, tumor growth in the hTERT-shRNA group was slightly suppressed when no irradiation was applied, and TUNEL results revealed that the apoptosis index in this group was slightly increased. Moreover, HE staining results also found that the necrotic area in the hTERT-shRNA group was slightly increased. Further research discovered that the growth of tumors in each group was notably suppressed after exposure to irradiation. However, the degree of growth suppression in the hTERT-shRNA group was more obvious, as could be observed from the tumor growth curve and tumor growth rate after irradiation, and the apoptosis index in that group was also markedly increased. These results strongly suggested that hTERT silencing could not only promote apoptosis and suppress proliferation but also enhance the radiosensitivity of CNE-2R cells *in vivo*.

Previous studies found that silencing hTERT could eliminate the CSC phenotype [18–20]. Our research results also suggest that the expression of stem cell-related proteins in CNE-2R cells was down-regulated both *in vitro* and *in vivo* after silencing hTERT, and the CSC-like characteristics were decreased. An increasing number of studies have verified that radioresistance of tumors is associated with CSCs [42–45]. Krause et al. proposed that CSCs mediated tumors to develop radioresistance through multiple mechanisms [46, 47]. Similarly, studies on NPC also indicated that CSC-like cells displayed obvious radioresistance [48–51]. Moreover, some studies reported that silencing the telomeric repeat binding factor-2 (TRF2) gene could enhance the radiosensitivity of telomerase-immortalized human mesenchymal stem cells [52, 53]. Therefore, we believe that the enhanced radiosensitivity of CNE-2R cells after silencing hTERT might be related to the reduced CSC-like characteristics. In addition, we discovered that silencing hTERT could significantly decrease telomerase activity. Some studies proved that suppressing telomerase activity enhanced the radiosensitivity of multiple tumors [23–26]. Berardinelli suggested that targeting telomere/telomerase was one of the most promising methods to enhance the radiosensitivity of tumor cells [54]. Some scholars found that telomerase is highly expressed in CSCs [11, 12, 25], which was essential for the self-renewal, progression and immortalization of CSCs [13]. Consequently, we speculate that silencing hTERT may suppress telomerase activity through the hTERT/telomerase pathway, which can attenuate the CSC-like characteristics of CNE-2R cells, thus enhancing their radiosensitivity.

Additionally, our western blot results showed that, compared with that in NC cells and CNE-2R cells, the total β-catenin protein expression in hTERT-shRNA cells showed no significant change. However, IHC results demonstrated that β-catenin protein expression in the hTERT-shRNA group was mainly located at the membrane and cytoplasm and that β-catenin protein expression in some cells of the NC and CNE-2R groups could be located in the nucleus. Such interesting findings indicated that silencing hTERT might not affect the total β-catenin protein expression but would change its expression localization. There might be a regulatory relationship between hTERT and the Wnt/β-catenin pathway, but how they interact still remains controversial [55–58]. β-catenin plays an important role in maintaining the NPC CSC phenotype, which confirms that the Wnt/β-catenin pathway plays a regulatory role in CSCs [59, 60]. Our previous study also found that CNE-2R cells highly expressed β-catenin protein compared with parental CNE-2 cells [10]. Therefore, we speculate that the Wnt/β-catenin pathway may be involved in the regulation of radiosensitivity of CNE-2R cells by hTERT, which is our next research focus.

In conclusion, our study showed that silencing hTERT could enhance the radiosensitivity of CNE-2R cells both *in vitro* and *in vivo*. Moreover, we also discovered that silencing hTERT could reduce telomerase activity and suppress the CSC-like characteristics of CNE-2R cells. Overall, the down-regulation of hTERT could inhibit telomerase activity, which could then affect the CSC-like characteristics of CNE-2R cells, finally enhancing the radiosensitivity of CNE-2R cells. These results also revealed that the hTERT/telomerase pathway might become a therapeutic target for radioresistance in NPC.

## MATERIALS AND METHODS

### Cell lines and cells culture

The poorly differentiated NPC cell line CNE-2 was purchased from Fudan University Shanghai Cancer Center (Shanghai, China). The CNE-2 cells were tested by Short Tandem Repeat profiling on March 15, 2018. The radioresistant cell line CNE-2R was established by subjecting CNE-2 to fractional irradiation in our previous study [9]. The cells were cultured in DMEM containing 10% fetal bovine serum (Gibco, USA), 100 U/mL penicillin and 0.1 mg/mL streptomycin (Solarbio, China) in a saturated humidity incubator containing 5% CO_2_ at 37° C.

### Construction of lentiviral vector and infection of CNE-2R cells

The RNAi target sequence of the hTERT gene (Accession No.: NM_198253.2) was designed as 5'-AAGTTGCAAAGCATTGGAA-3' according to the RNAi sequence design principle, while the RNAi negative control scramble sequence was designed as 5'-TTCTCCGAACGTGTCACGT-3'. The lentiviral vector was constructed by Shanghai Genechem Co., Ltd. (Shanghai, China) using the GV493 vector according to the vector element of hU6-MCS-CBh-gcGFP-IRES-puromycin. CNE-2R cells were prepared into suspension at a density of 5×10^4^ cells/mL with complete medium. Then, 2 mL/well suspension (approximately 10^5^ cells) was seeded into the 6-well culture plate. After 24 h of culture, the cells had grown to approximately 2×10^5^ cells/well, the medium was discarded, and 1 mL Enhanced Infection Solution containing 5 μg/mL Polybrene (Genechem, China) was added. The hTERT-shRNA and negative control (NC) cells were obtained by using target gene and NC lentiviral vectors (MOI=30) to infect cells, respectively. Ninety-six hours after infection, the infection rate was observed using an inverted fluorescence microscope (Olympus Corporation, Japan).

### Real-time quantitative PCR (qPCR)

Total RNA was extracted from cells using TRIzol reagent (Invitrogen, USA), and cDNA was obtained through reverse transcription using the PrimeScript RT reagent Kit (Takara, Japan). The mRNA expression of the hTERT gene was detected using the SYBR Premix Ex TaqTM II kit (Takara, Japan), with GAPDH as the reference gene. The gene primers were as follows (5'-3'): hTERT-F: CTCCCATTTCATCAGCAAGTTT, hTERT-R: CTTGGCTTTCAGGATGGAGTAG; GAPDH-F: CAGGAGGCATTGCTGATGAT, and GAPDH-R: GAAGGCTGGGGCTCATTT. The relative mRNA expression was calculated by the 2^-ΔΔCt^ method.

### Colony-formation assay

CNE-2R cells, NC cells and hTERT-shRNA cells were seeded into six-well plates at cell numbers of 200, 200, 400, 600 and 1000, respectively. The cells were then cultured to adherence, followed by 6 MV-X ray irradiation at different doses (0, 2, 4, 6 and 8 Gy for each cell number). After the completion of irradiation, the cells were further cultured for 14 days, followed by 4% paraformaldehyde fixation for 30 min and Giemsa staining for 30 min. The number of colonies containing ≥ 50 cells at different irradiation doses was counted. Then, the dose-survival curves were fitted using the multi-target single-hit model y=1-(1-exp(-k*x))^N, and the radiobiological parameters D_0_, D_q_ and SF_2_ were calculated, where D_0_=1/K and D_q_=lnN* D_0_.

### CCK-8 assay

First, the proliferation capacity of cells in each group was detected. In brief, cells were seeded into 96-well plates at a cell number of 2×10^3^ /well, and the cell viability was detected on days 0 (after cell adherence), 1, 2, 3, 4 and 5. Subsequently, the radiosensitivity of cells in each group was also detected. Similarly, cells were seeded into 96-well plates (2×10^3^ cells/well), and each group of cells was divided into 5 subgroups according to the different irradiation doses (0, 2, 4, 6 and 8 Gy). After adherence, cells were exposed to 6 MV-X ray, and cell viability was detected 48 h after irradiation. Cell viability was assessed using the Cell Counting Kit-8 (CCK-8) (Dojindo, Japan) assay according to the manufacturer's instructions. The optical density (OD) value at a wavelength of 450 nm was detected using the Microplate Reader (Thermo, USA). Finally, the radiosensitivity of cells in each group was evaluated by the survival fraction (SF), which was calculated as follows: SF=OD/OD_0Gy_.

### Apoptosis rate analysis using flow cytometry

The apoptosis rate was detected using the Annexin V-APC/7-AAD kit (BD, USA). Cells were exposed to 6 MV-X ray at doses of 0 and 4 Gy, and all cells in each group were collected 48 h after irradiation. Then, the cells were labelled with 5 μL Annexin V-APC and 5 μL 7-AAD according to the manufacturer’s instructions. Apoptotic cells were analyzed by the flow cytometry system (BD FACSCalibur, USA) within 1 h.

### Western blot assay

Total proteins of CNE-2R, NC and hTERT-shRNA cells were extracted using RIPA lysis buffer, after which 50 μg total proteins were separated by SDS-PAGE electrophoresis, and the electrophoresis products were transferred onto PVDF membranes through the wet transfer method. Afterwards, the PVDF membranes were blocked with 5% skim milk for 1.5 h and washed with 1×TBST. Then, the membranes were incubated with primary antibodies against hTERT (Abcam, UK), CD133 (Wanleibio, China), β-catenin, Sox2, Nanog, Bmi1, Oct4, Survivin, Caspase 3, GAPDH and β-actin (CST, USA), respectively, followed by slow shaking on a shaking table at 4° C overnight. The PVDF membranes were taken out and washed with 1×TBST, followed by incubation with secondary antibody IgG HRP (CST, USA) at room temperature (RT) for 1 h and washing with 1×TBST. The images were collected using the Gel Imaging System (Bio-Rad, USA).

### Telomerase activity assay

Telomerase activity was measured using the TeloTAGGG Telomerase PCR ELISA kit (Roche, Switzerland). Telomerase activity measurement was performed as previously described [10]. An RNase-treated extract was used as a negative control, and 293 cell extract was used as a positive control.

### Xenograft model

BALB/c nude mice were purchased from the Laboratory Animal Center of Guangxi Medical University (Nanning, China). CNE-2R, NC and hTERT-shRNA cells were subcutaneously injected into the left groin of nude mice at a dose of 1×10^7^, and 8 nude mice were used in each group. Each nude mouse was labeled with the ear tag. After tumor formation, the long (a) and short (b) diameters of the tumors were measured with an electronic vernier caliper every 3 days, and the tumor volume was calculated according to the formula V=ab^2^/2. When the long tumor diameter had reached approximately 10 mm, the nude mice in each group were randomly divided into 2 subgroups, with 4 in each subgroup, and received 6 MV-X ray irradiation at doses of 0 and 8 Gy. Afterwards, the tumors were further observed for 15 days, and the tumor growth rate was calculated as follows: growth rate=(V_t_-V_0_)/V_0_, where V_t_ represented the volume at each measurement after irradiation, and V_0_ stood for the tumor volume measured before irradiation. The procedures involving animals and their care were approved by the Laboratory Animal Care and Use Committee of the Guangxi Medical University.

### TdT-mediated dUTP Nick-End Labeling (TUNEL) assay

Apoptotic cells in xenografts were detected using the In Situ Cell Death Detection Kit (TMR red) (Roche, Switzerland). Briefly, the paraffin sections were deparaffinized and hydrated. Microwave-based antigen retrieval was performed in citrate buffer solution (pH=6.0) for 5 min. The enzyme solution (TdT) and label solution (dUTP) in the kit were mixed at a ratio of 1:9 (v/v) to obtain the Reaction Solution. One hundred microliters of reaction solution was added to cover the tissue and incubated at 37° C for 1 h in the dark. DAPI staining solution (Beyotime, China) was added and incubated for 10 min at RT in the dark. Subsequently, the sections were mounted using anti-fluorescence quenching medium (Beyotime, China). Apoptotic cells were counted using a fluorescence microscope (Olympus Corporation, Japan) in 10 randomly selected high-power fields (200×), and the apoptosis index was calculated as a percentage of at least 1,000 scored cells.

### Immunohistochemistry (IHC)

Paraffin sections from the unirradiated (0 Gy) xenografts were deparaffinized and hydrated according to standard protocols. High-pressure heating antigen retrieval was performed in citrate buffer solution (pH=6.0). Endogenous peroxidase activity was blocked with 3% H_2_O_2_. Then, nonspecific antigens were blocked with 3% BSA. The primary antibodies were applied at 4° C overnight, followed by a biotinylated secondary antibody and HRP-labeled streptavidin (ZSGB-BIO, China). The peroxidase reaction was carried out with a DAB kit (ZSGB-BIO, China). The sections were counterstained with hematoxylin, mounted with neutral balsam, and observed under a light microscope (Olympus Corporation, Japan). The results were analyzed according to the methods employed in our previous study [41].

### Statistical analysis

GraphPad Prism 5.0 (GraphPad Software, San Diego, CA, USA) or SPSS 20.0 (IBM, Armonk, NY, USA) software was used for statistical analyses, and the data are expressed as the mean ± standard deviation (SD). The statistical significance of the differences in the *in vitro* experiments was determined using two-tailed Student’s t-test or one-way ANOVA. Moreover, differences in tumor growth among different groups were assessed by ANOVA with a repeated measurement module. A two-tailed difference of P<0.05 was considered statistically significant.

## References

[1] Chen W, Zheng R, Baade PD, Zhang S, Zeng H, Bray F, Jemal A, Yu XQ, He J. Cancer statistics in China, 2015. CA Cancer J Clin. 2016; 66:115–32. 10.3322/caac.21338 26808342

[2] Cao SM, Simons MJ, Qian CN. The prevalence and prevention of nasopharyngeal carcinoma in China. Chin J Cancer. 2011; 30:114–19. 10.5732/cjc.010.10377 21272443 PMC4013340

[3] Chan AT, Grégoire V, Lefebvre JL, Licitra L, Hui EP, Leung SF, Felip E, and EHNS–ESMO–ESTRO Guidelines Working Group. Nasopharyngeal cancer: EHNS-ESMO-ESTRO clinical practice guidelines for diagnosis, treatment and follow-up. Ann Oncol. 2012 (Suppl 7); 23:vii83–85. 10.1093/annonc/mds266 22997460

[4] Pan JJ, Ng WT, Zong JF, Lee SW, Choi HC, Chan LL, Lin SJ, Guo QJ, Sze HC, Chen YB, Xiao YP, Kan WK, O’Sullivan B, et al. Prognostic nomogram for refining the prognostication of the proposed 8th edition of the AJCC/UICC staging system for nasopharyngeal cancer in the era of intensity-modulated radiotherapy. Cancer. 2016; 122:3307–15. 10.1002/cncr.30198 27434142 PMC5524130

[5] Liu X, Tang LL, Du XJ, Li WF, Chen L, Zhou GQ, Guo R, Liu Q, Sun Y, Ma J. Changes in disease failure risk of nasopharyngeal carcinoma over time: analysis of 749 patients with long-term follow-up. J Cancer. 2017; 8:455–59. 10.7150/jca.17104 28261347 PMC5332897

[6] Sun X, Su S, Chen C, Han F, Zhao C, Xiao W, Deng X, Huang S, Lin C, Lu T. Long-term outcomes of intensity-modulated radiotherapy for 868 patients with nasopharyngeal carcinoma: an analysis of survival and treatment toxicities. Radiother Oncol. 2014; 110:398–403. 10.1016/j.radonc.2013.10.020 24231245

[7] Zhu H, Zhu X, Cheng G, Zhou M, Lou W. Downregulation of microRNA-21 enhances radiosensitivity in nasopharyngeal carcinoma. Exp Ther Med. 2015; 9:2185–89. 10.3892/etm.2015.2403 26136957 PMC4473658

[8] Feng XP, Yi H, Li MY, Li XH, Yi B, Zhang PF, Li C, Peng F, Tang CE, Li JL, Chen ZC, Xiao ZQ. Identification of biomarkers for predicting nasopharyngeal carcinoma response to radiotherapy by proteomics. Cancer Res. 2010; 70:3450–62. 10.1158/0008-5472.CAN-09-4099 20406978

[9] Guo Y, Zhu XD, Qu S, Li L, Su F, Li Y, Huang ST, Li DR. Identification of genes involved in radioresistance of nasopharyngeal carcinoma by integrating gene ontology and protein-protein interaction networks. Int J Oncol. 2012; 40:85–92. 10.3892/ijo.2011.1172 21874234

[10] Chen KH, Guo Y, Li L, Qu S, Zhao W, Lu QT, Mo QY, Yu BB, Zhou L, Lin GX, Sun YC, Zhu XD. Cancer stem cell-like characteristics and telomerase activity of the nasopharyngeal carcinoma radioresistant cell line CNE-2R. Cancer Med. 2018; 7:4755–64. 10.1002/cam4.1729 30105829 PMC6144248

[11] Zhang X, Komaki R, Wang L, Fang B, Chang JY. Treatment of radioresistant stem-like esophageal cancer cells by an apoptotic gene-armed, telomerase-specific oncolytic adenovirus. Clin Cancer Res. 2008; 14:2813–23. 10.1158/1078-0432.CCR-07-1528 18451249 PMC2387204

[12] Shay JW, Wright WE. Telomeres and telomerase in normal and cancer stem cells. FEBS Lett. 2010; 584:3819–25. 10.1016/j.febslet.2010.05.026 20493857 PMC3370416

[13] Ju Z, Rudolph KL. Telomeres and telomerase in cancer stem cells. Eur J Cancer. 2006; 42:1197–203. 10.1016/j.ejca.2006.01.040 16644207

[14] Daniel M, Peek GW, Tollefsbol TO. Regulation of the human catalytic subunit of telomerase (hTERT). Gene. 2012; 498:135–46. 10.1016/j.gene.2012.01.095 22381618 PMC3312932

[15] Aschacher T, Wolf B, Enzmann F, Kienzl P, Messner B, Sampl S, Svoboda M, Mechtcheriakova D, Holzmann K, Bergmann M. LINE-1 induces hTERT and ensures telomere maintenance in tumour cell lines. Oncogene. 2016; 35:94–104. 10.1038/onc.2015.65 25798839

[16] Harley CB. Telomerase and cancer therapeutics. Nat Rev Cancer. 2008; 8:167–79. 10.1038/nrc2275 18256617

[17] Dikmen ZG, Gellert GC, Jackson S, Gryaznov S, Tressler R, Dogan P, Wright WE, Shay JW. *In vivo* inhibition of lung cancer by GRN163L: a novel human telomerase inhibitor. Cancer Res. 2005; 65:7866–73. 10.1158/0008-5472.CAN-05-1215 16140956

[18] Grandjenette C, Schnekenburger M, Gaigneaux A, Gérard D, Christov C, Mazumder A, Dicato M, Diederich M. Human telomerase reverse transcriptase depletion potentiates the growth-inhibitory activity of imatinib in chronic myeloid leukemia stem cells. Cancer Lett. 2020; 469:468–80. 10.1016/j.canlet.2019.11.017 31734352

[19] Hao J, Fan W, Li Y, Tang R, Tian C, Yang Q, Zhu T, Diao C, Hu S, Chen M, Guo P, Long Q, Zhang C, et al. Melatonin synergizes BRAF-targeting agent vemurafenib in melanoma treatment by inhibiting iNOS/hTERT signaling and cancer-stem cell traits. J Exp Clin Cancer Res. 2019; 38:48. 10.1186/s13046-019-1036-z 30717768 PMC6360719

[20] Feng X, Xu X, Xiao X, Zou K, Yu W, Wu J, Tang R, Gao Y, Hao J, Zhao X, Liao Y, Chen Y, Huang W, et al. NMI inhibits cancer stem cell traits by downregulating hTERT in breast cancer. Cell Death Dis. 2017; 8:e2783. 10.1038/cddis.2017.200 28492540 PMC5520720

[21] Zhang W, Xing L. RNAi gene therapy of SiHa cells via targeting human TERT induces growth inhibition and enhances radiosensitivity. Int J Oncol. 2013; 43:1228–34. 10.3892/ijo.2013.2051 23921425

[22] Wang W, Yang L, Hu L, Li F, Ren L, Yu H, Liu Y, Xia L, Lei H, Liao Z, Zhou F, Xie C, Zhou Y. Inhibition of UBE2D3 expression attenuates radiosensitivity of MCF-7 human breast cancer cells by increasing hTERT expression and activity. PLoS One. 2013; 8:e64660. 10.1371/journal.pone.0064660 23741361 PMC3669415

[23] Wu X, Zhang J, Yang S, Kuang Z, Tan G, Yang G, Wei Q, Guo Z. Telomerase antagonist imetelstat increases radiation sensitivity in esophageal squamous cell carcinoma. Oncotarget. 2017; 8:13600–19. 10.18632/oncotarget.14618 28099140 PMC5355123

[24] Yang L, Xu Z, Liu L, Luo X, Lu J, Sun L, Cao Y. Targeting EBV-LMP1 DNAzyme enhances radiosensitivity of nasopharyngeal carcinoma cells by inhibiting telomerase activity. Cancer Biol Ther. 2014; 15:61–68. 10.4161/cbt.26606 24145206 PMC3938524

[25] Wesbuer S, Lanvers-Kaminsky C, Duran-Seuberth I, Bölling T, Schäfer KL, Braun Y, Willich N, Greve B. Association of telomerase activity with radio- and chemosensitivity of neuroblastomas. Radiat Oncol. 2010; 5:66. 10.1186/1748-717X-5-66 20642823 PMC2917444

[26] Ji XM, Xie CH, Fang MH, Zhou FX, Zhang WJ, Zhang MS, Zhou YF. Efficient inhibition of human telomerase activity by antisense oligonucleotides sensitizes cancer cells to radiotherapy. Acta Pharmacol Sin. 2006; 27:1185–91. 10.1111/j.1745-7254.2006.00417.x 16923339

[27] Liu T, Li W, Lu W, Chen M, Luo M, Zhang C, Li Y, Qin G, Shi D, Xiao B, Qiu H, Yu W, Kang L, et al. RBFOX3 promotes tumor growth and progression via hTERT signaling and predicts a poor prognosis in hepatocellular carcinoma. Theranostics. 2017; 7:3138–54. 10.7150/thno.19506 28839469 PMC5566111

[28] Chen L, Chen C, Chen W, Li K, Chen X, Tang X, Xie G, Luo X, Wang X, Liang H, Yu S. Biodegradable black phosphorus nanosheets mediate specific delivery of hTERT siRNA for synergistic cancer therapy. ACS Appl Mater Interfaces. 2018; 10:21137–48. 10.1021/acsami.8b04807 29882656

[29] Chen P, Gu WL, Gong MZ, Wang J, Li DQ. shRNA-mediated silencing of hTERT suppresses proliferation and promotes apoptosis in osteosarcoma cells. Cancer Gene Ther. 2017; 24:325–32. 10.1038/cgt.2017.22 28799566

[30] Shi YA, Zhao Q, Zhang LH, Du W, Wang XY, He X, Wu S, Li YL. Knockdown of hTERT by siRNA inhibits cervical cancer cell growth *in vitro* and *in vivo*. Int J Oncol. 2014; 45:1216–24. 10.3892/ijo.2014.2493 24920549

[31] Li Y, Pan G, Chen Y, Yang Q, Hao T, Zhao L, Zhao L, Cong Y, Diao A, Yu P. Inhibitor of the human telomerase reverse trancriptase (hTERT) gene promoter induces cell apoptosis via a mitochondrial-dependent pathway. Eur J Med Chem. 2018; 145:370–78. 10.1016/j.ejmech.2017.12.077 29335203

[32] Zhao X, Zhang C, Le Z, Zeng S, Pan C, Shi J, Wang J, Zhao X. Telomerase reverse transcriptase interference synergistically promotes tumor necrosis factor-related apoptosis-inducing ligand-induced oral squamous cell carcinoma apoptosis and suppresses proliferation *in vitro* and *in vivo*. Int J Mol Med. 2018; 42:1283–94. 10.3892/ijmm.2018.3721 29901096 PMC6089774

[33] Pal D, Sharma U, Singh SK, Kakkar N, Prasad R. Inhibition of hTERT expression by MAP kinase inhibitor induces cell death in renal cell carcinoma. Urol Oncol. 2017; 35:401–08. 10.1016/j.urolonc.2017.01.019 28215740

[34] Wu XQ, Huang C, He X, Tian YY, Zhou DX, He Y, Liu XH, Li J. Feedback regulation of telomerase reverse transcriptase: new insight into the evolving field of telomerase in cancer. Cell Signal. 2013; 25:2462–68. 10.1016/j.cellsig.2013.08.009 23993966

[35] Song YJ, Han JB, Chen SM, Xiao BK, Chen C. Effect of Adv Vector-mediated shRNA Targeting hTERT on Proliferation and Apoptosis of Nasopharyngeal Carcinoma Cells. Canc Res Prev Treat. 2011; 38:1351–55.

[36] Kang X, Wang F, Lan X, Li X, Zheng S, Lv Z, Zhuang Y, Zhao Y, Zhou S. Lentivirus-mediated shRNA targeting CNN2 inhibits hepatocarcinoma *in vitro* and *in vivo*. Int J Med Sci. 2018; 15:69–76. 10.7150/ijms.21113 29333089 PMC5765741

[37] Ikegami A, Teixeira LF, Braga MS, Dias MH, Lopes EC, Bellini MH. Knockdown of NF-κB1 by shRNA inhibits the growth of renal cell carcinoma *in vitro* and *in vivo*. Oncol Res. 2018; 26:743–51. 10.3727/096504017X15120379906339 29212573 PMC7844753

[38] Yuan Q, Yu H, Chen J, Song X, Sun L. Knockdown of pyruvate kinase type M2 suppresses tumor survival and invasion in osteosarcoma cells both *in vitro* and *in vivo*. Exp Cell Res. 2018; 362:209–16. 10.1016/j.yexcr.2017.11.020 29155364

[39] Cheng Q, Xu X, Jiang H, Xu L, Li Q. Knockdown of long non-coding RNA XIST suppresses nasopharyngeal carcinoma progression by activating miR-491-5p. J Cell Biochem. 2018; 119:3936–44. 10.1002/jcb.26535 29219216

[40] Shang QY, Wu CS, Gao HR. Effects of DCK knockdown on proliferation, apoptosis and tumorigenicity *in vivo* of cervical cancer HeLa cells. Cancer Gene Ther. 2017; 24:367–72. 10.1038/cgt.2017.31 28820179

[41] Yu BB, Lin GX, Li L, Qu S, Liang ZG, Chen KH, Zhou L, Lu QT, Sun YC, Zhu XD. Cofilin-2 acts as a marker for predicting radiotherapy response and is a potential therapeutic target in nasopharyngeal carcinoma. Med Sci Monit. 2018; 24:2317–29. 10.12659/msm.909832 29664897 PMC5921956

[42] Qi XS, Pajonk F, McCloskey S, Low DA, Kupelian P, Steinberg M, Sheng K. Radioresistance of the breast tumor is highly correlated to its level of cancer stem cell and its clinical implication for breast irradiation. Radiother Oncol. 2017; 124:455–61. 10.1016/j.radonc.2017.08.019 28923575 PMC6128144

[43] Da C, Wu L, Liu Y, Wang R, Li R. Effects of irradiation on radioresistance, HOTAIR and epithelial-mesenchymal transition/cancer stem cell marker expression in esophageal squamous cell carcinoma. Oncol Lett. 2017; 13:2751–57. 10.3892/ol.2017.5774 28454462 PMC5403525

[44] Koch U, Krause M, Baumann M. Cancer stem cells at the crossroads of current cancer therapy failures—radiation oncology perspective. Semin Cancer Biol. 2010; 20:116–24. 10.1016/j.semcancer.2010.02.003 20219680

[45] Chen YW, Chen KH, Huang PI, Chen YC, Chiou GY, Lo WL, Tseng LM, Hsu HS, Chang KW, Chiou SH. Cucurbitacin I suppressed stem-like property and enhanced radiation-induced apoptosis in head and neck squamous carcinoma—derived CD44(+)ALDH1(+) cells. Mol Cancer Ther. 2010; 9:2879–92. 10.1158/1535-7163.MCT-10-0504 21062915

[46] Krause M, Yaromina A, Eicheler W, Koch U, Baumann M. Cancer stem cells: targets and potential biomarkers for radiotherapy. Clin Cancer Res. 2011; 17:7224–29. 10.1158/1078-0432.CCR-10-2639 21976536

[47] Krause M, Dubrovska A, Linge A, Baumann M. Cancer stem cells: radioresistance, prediction of radiotherapy outcome and specific targets for combined treatments. Adv Drug Deliv Rev. 2017; 109:63–73. 10.1016/j.addr.2016.02.002 26877102

[48] Zhang G, Wang W, Yao C, Zhang S, Liang L, Han M, Ren J, Qi X, Zhang X, Wang S, Li L. Radiation-resistant cancer stem-like cell properties are regulated by PTEN through the activity of nuclear β-catenin in nasopharyngeal carcinoma. Oncotarget. 2017; 8:74661–72. 10.18632/oncotarget.20339 29088815 PMC5650370

[49] Yang CF, Peng LX, Huang TJ, Yang GD, Chu QQ, Liang YY, Cao X, Xie P, Zheng LS, Huang HB, Cai MD, Huang JL, Liu RY, et al. Cancer stem-like cell characteristics induced by EB virus-encoded LMP1 contribute to radioresistance in nasopharyngeal carcinoma by suppressing the p53-mediated apoptosis pathway. Cancer Lett. 2014; 344:260–71. 10.1016/j.canlet.2013.11.006 24262659

[50] Su J, Xu XH, Huang Q, Lu MQ, Li DJ, Xue F, Yi F, Ren JH, Wu YP. Identification of cancer stem-like CD44+ cells in human nasopharyngeal carcinoma cell line. Arch Med Res. 2011; 42:15–21. 10.1016/j.arcmed.2011.01.007 21376257

[51] Wang J, Guo LP, Chen LZ, Zeng YX, Lu SH. Identification of cancer stem cell-like side population cells in human nasopharyngeal carcinoma cell line. Cancer Res. 2007; 67:3716–24. 10.1158/0008-5472.CAN-06-4343 17440084

[52] Serakinci N, Mega Tiber P, Orun O. Chromatin modifications of hTERT gene in hTERT-immortalized human mesenchymal stem cells upon exposure to radiation. Eur J Med Genet. 2018; 61:288–93. 10.1016/j.ejmg.2017.12.014 29288791

[53] Orun O, Tiber PM, Serakinci N. Partial knockdown of TRF2 increase radiosensitivity of human mesenchymal stem cells. Int J Biol Macromol. 2016; 90:53–58. 10.1016/j.ijbiomac.2015.10.072 26598048

[54] Berardinelli F, Coluzzi E, Sgura A, Antoccia A. Targeting telomerase and telomeres to enhance ionizing radiation effects in *in vitro* and *in vivo* cancer models. Mutat Res. 2017; 773:204–19. 10.1016/j.mrrev.2017.02.004 28927529

[55] Park JI, Venteicher AS, Hong JY, Choi J, Jun S, Shkreli M, Chang W, Meng Z, Cheung P, Ji H, McLaughlin M, Veenstra TD, Nusse R, et al. Telomerase modulates Wnt signalling by association with target gene chromatin. Nature. 2009; 460:66–72. 10.1038/nature08137 19571879 PMC4349391

[56] Hoffmeyer K, Raggioli A, Rudloff S, Anton R, Hierholzer A, Del Valle I, Hein K, Vogt R, Kemler R. Wnt/β-catenin signaling regulates telomerase in stem cells and cancer cells. Science. 2012; 336:1549–54. 10.1126/science.1218370 22723415

[57] Liu Z, Li Q, Li K, Chen L, Li W, Hou M, Liu T, Yang J, Lindvall C, Björkholm M, Jia J, Xu D. Telomerase reverse transcriptase promotes epithelial-mesenchymal transition and stem cell-like traits in cancer cells. Oncogene. 2013; 32:4203–13. 10.1038/onc.2012.441 23045275

[58] Jung JH, Kang KW, Kim J, Hong SC, Park Y, Kim BS. CXCR2 inhibition in human pluripotent stem cells induces predominant differentiation to mesoderm and endoderm through repression of mTOR, β-catenin, and hTERT activities. Stem Cells Dev. 2016; 25:1006–19. 10.1089/scd.2015.0395 27188501 PMC4931345

[59] Jiang R, Niu X, Huang Y, Wang X. Β-catenin is important for cancer stem cell generation and tumorigenic activity in nasopharyngeal carcinoma. Acta Biochim Biophys Sin (Shanghai). 2016; 48:229–37. 10.1093/abbs/gmv134 26849897 PMC4885127

[60] Cheng Y, Cheung AK, Ko JM, Phoon YP, Chiu PM, Lo PH, Waterman ML, Lung ML. Physiological β-catenin signaling controls self-renewal networks and generation of stem-like cells from nasopharyngeal carcinoma. BMC Cell Biol. 2013; 14:44. 10.1186/1471-2121-14-44 24073846 PMC3819748

